# Frenemy: adaptive temperate phage_SAP_1432 supports *Staphylococcus aureus* survival in changing temperatures

**DOI:** 10.1128/spectrum.02271-24

**Published:** 2025-06-23

**Authors:** Ting-ting Liu, Peng-cheng Gao, Jie-wen Cui, Wu-bin Wang, Fu-ying Zheng, Xue-rui Li, Yue-feng Chu

**Affiliations:** 1 State Key Laboratory for Animal Disease Control and Prevention, College of Veterinary Medicine, Lanzhou University, Lanzhou Veterinary Research Institute, Chinese Academy of Agricultural Sciences 12426, Lanzhou, Gansu, China; Università degli Studi di Napoli Federico II, Naples, Italy

**Keywords:** *Staphylococcus aureus*, heat-resistant temperate phage, temperature, thermal performance curve, lysogeny

## Abstract

It is important to understand how the presence of pathogens could benefit the survival of their hosts during climate change. In this study, we investigated the influence of the heat-resistant temperate phage phage_SAP_1432 on its bacterial host *Staphylococcus aureus* Q1432 at different temperatures. Thermal host performance curves at a multiplicity of infection of 0.001 showed that the phage killed the bacterium efficiently from 20 to 39°C and from 47 to 50°C. However, the phage resulted in a 41.2% increase in the maximal growth rate for the host bacterium from 1.53 (95% confidence interval [CI]: 1.42–1.63) in the absence of phage_SAP_1432 to 2.16 (95% CI: 2.02–2.37) in its presence, along with a shift in the optimum temperature from 41.0°C to 44.1°C. In the absence of phage, the maximum survival temperature of *S. aureus* Q1432 was 51°C, but after co-culture with phage_SAP_1432, a few bacteria survived at 55°C–80°C. Lysogenesis occurred more easily at lower temperatures; the percentage of lysogenesis increased with phage concentration, while the maximum growth rate of the host decreased. Our findings provide new evidence that heat-resistant temperate phages can benefit the survival of their bacterial host at specific temperatures, giving a new perspective on the effects of co-evolution of heat-resistant temperate phages and bacterial hosts in an era of global climate change.

**IMPORTANCE:**

Understanding pathogen–host interactions is crucial for predicting climate change impacts on microbial ecosystems. This study examined the heat-resistant temperate phage_SAP_1432 and its effects on *Staphylococcus aureus* Q1432 at various temperatures. Phage_SAP_1432 enhanced the thermal performance and survival at high temperatures of its host at a low MOI. This mutual benefit demonstrates the adaptive advantages phages provide in changing thermal environments. As global temperatures rise, such phage–host interactions may play a critical role in microbial survival and evolution. Our research highlights the potential for phages to act as allies, offering a new perspective on the co-evolution of heat-resistant temperate phages and their bacterial hosts.

## INTRODUCTION

Global warming is a severe problem; even a 1°C–2°C increase in global temperature could severely impact the survival of many species (1). Because of this, it is of great importance to understand how temperature changes affect biological systems as well as the role of adaptation in population persistence. In addition to studies examining the adaptation of single populations in coping with climate change (2, 3), much attention has been paid to the role of antagonistic co-evolution between hosts and pathogens concerning population persistence in the face of temperature changes (4, 5).

Bacteriophages propagate by lysing bacterial cells and play important roles in bacterial physiology and evolution (6, 7). Lytic phages attack and kill their hosts, reshaping bacterial population dynamics (8, 9). Temperate phages not only shape host evolution by affecting population dynamics through lysis but can also integrate their DNA into host chromosomes in a process called lysogeny (8, 10). In natural environments, the physiology, ecology, and evolution of bacterial hosts and phages are likely to be shaped both by host-phage interactions as well as by the physical environment, of which temperature is an important factor (11).

Several studies have explored how temperature influences host–pathogen interactions in phage–bacteria co-evolution (12, 13). Duncan et al. (14) investigated the effects of fluctuating environmental conditions on the antagonistic co-evolution between *Pseudomonas fluorescens* SBW25 and its lytic bacteriophage SBWΦ2. Co-evolution stalled during periods of high temperature under both intermediate- and low-frequency temperature fluctuations, generating temporary co-evolutionary cold spots. Additionally, temperature variation significantly affected population density (14). Padfield et al. (13) investigated how temperature changes the bacterium *P. fluorescens* thermal performance curves (TPCs), which describe the relationship between temperature and the biological performance of an organism; TPCs can help predict changes in species interactions and provide insights into the ecological and evolutionary mechanisms driving microbial host–pathogen dynamics. In the presence of SBWΦ2, host TPCs were altered, leading to a reduced maximum growth rate primarily driven by differences in thermal tolerance and the temperature-dependent costs of evolved resistance (13). While lytic phages have been extensively studied in this context, temperate phages can also play a crucial role in shaping bacterial communities. Understanding how temperate phages influence bacterial evolution across different temperatures remains an important avenue for future research.

In this study, we conducted an experimental co-evolution assay using a heat-resistant temperate phage (phage_SAP_1432) and its bacterial host (*Staphylococcus aureus* Q1432) to assess the impact of the phage on bacterial survival across different temperatures. We aimed to investigate whether temperate phages alter the TPC of their hosts differently from lytic phages and to determine whether heat-resistant temperate phages benefit the survival of their bacterial host at specific temperatures.

## MATERIALS AND METHODS

### Bacteria and phage

*S. aureus Q1432*, *S. aureus* Q1622, *S. aureus* Q14261, and *S. aureus* Q14098 (GenBank number: PV256628, PV256629, PV256630, and PV256631) were isolated by our research group from the milk of cows with mastitis on a dairy farm in Qinghai Province, China. Milk samples were evenly spread onto Luria-Bertani (LB) agar plates and incubated overnight at 37°C. Individual colonies were then selected and cultured in LB broth. Colonies were confirmed as S. *aureus* through 16S rDNA gene sequencing. Drug sensitivity and heat resistance tests revealed that *S. aureus* Q1432 was resistant to both penicillin G and vancomycin; *S. aureus* Q1622 and *S. aureus* Q14261*S* were resistant to penicillin G, kanamycin, and vancomycin, while *S. aureus* Q14098 was resistant to penicillin G, tetracycline, and vancomycin. Heat resistance tests showed that *S. aureus* Q1432 could not survive after 1 h of incubation at >51°C, while *S. aureus* Q1622, *S. aureus* Q14261, and *S. aureus* Q14098 were all unable to grow after 1 h of incubation at >50°C.

Phage_SAP_1432 was isolated by our research group from sewage collected from a dairy farm in Xining, Qinghai Province, China. For phage isolation, 5 mL of sewage was mixed with 7.5 mL of phage buffer under sterile conditions. Subsequently, 1 mL of *S. aureus* Q1432 culture, grown to the logarithmic phase (optical density at 600 nm [OD_600_] = 0.6–0.8), was added to the mixture. The culture was incubated at 37°C with shaking at 160 rpm for 24 h. After incubation, the mixed culture was filtered using a 0.22 µm sterile filter to remove bacterial cells, and 1 mL of filtrate was mixed with 0.5 mL of host bacteria and incubated at 37°C for 15–20 min for phage adsorption. This mixture was combined with 4 mL of 0.7% molten LB agar and poured onto LB agar plates. The plates were incubated at 37°C for 24 h, after which the presence of plaques was observed, indicating successful phage isolation.

### Biological characteristics of phage_SAP_1432

The biological characteristics of phage _SAP_1432 were determined, including the optimal multiplicity of infection (MOI), one-step growth curve, thermal stability, and pH stability. To determine the optimal MOI, *S. aureus* Q1432 was cultured to the early logarithmic phase (OD_600_ = 0.2-0.4). The phage suspension was diluted and mixed with the bacterial culture at different MOIs (0.001, 0.01, 0.1, 1, 10, and 100). After incubation at 37°C for 10 min, the mixture was centrifuged at 8,000 × *g* for 5 min. The pellet was resuspended in 10 mL of LB broth and incubated at 37°C with shaking at 200 rpm for 5 h. The culture was then centrifuged again at 8,000 × *g* for 10 min, and the supernatant was filtered through a 0.22 µm membrane. Phage titers were determined using the double-layer agar method previously described and calculated after overnight incubation. The MOI that resulted in the highest phage production was considered optimal. The one-step growth curve was determined according to a previous report with minor modifications (15). Briefly, 1 mL of *S. aureus* Q1432 culture (OD_600_ = 0.5-0.6) was harvested and resuspended in 10 mL of fresh LB medium. The bacteria were then mixed with phage (MOI = 0.1) for 15 min at 37°C. The mixture was centrifuged at 8,000 × *g* for 10 min at 4°C, and pellets were resuspended in 10 mL of fresh LB medium that was preheated to 37°C. The suspension was incubated at 37°C while being shaken at 200 rpm. Samples were collected every 10 min for a total of 150 min, immediately diluted, and subsequently plated for phage titration by the double-agar layer method. The thermal stability of phage_SAP_1432 was assessed following the method described by Li et al. (15), with slight modifications. Briefly, phage suspensions were incubated in LB broth at 50°C, 60°C, 70°C, and 80°C for 60 min. pH tolerance was evaluated according to the procedure outlined by Hsieh et al. (16), with minor modifications. Phage suspensions were incubated at pH values of 2, 4, 6, 8, 10, and 12 for 1 h at room temperature (28°C). The number of surviving phage particles after stability experiments was determined by the double-layered agar plate method. All experiments were repeated three times.

### Sequencing and analysis of phage_SAP_1432 genome

Genomic DNA was extracted using the TIANamp Virus DNA/RNA Kit (Tiangen, Beijing, China) according to the manufacturer’s instructions. Phage genome sequencing was conducted by Huitong Biological Technology (Hangzhou, China). The sequencing library was prepared using the TruSeq DNA Sample Prep Kit (Illumina, San Diego, CA, USA), and sequencing was conducted on the Illumina NovaSeq 6000 platform using the TruSeq SBS Kit (Illumina, San Diego, CA, USA) with a read length of 150 bp. The genome sequence was assembled using SPAdes (version 3.6.2). Predicted gene protein sequences were subjected to Basic Local Alignment Search Tool (BLAST) alignment against multiple databases, including the nonredundant database, Clusters of Orthologous Genes, EggNOG, Kyoto Encyclopedia of Genes and Genomes, Swiss-Prot, and Gene Ontology, with an *e*-value threshold of ≤1 × 10^−5^ for functional annotation. Antibiotic resistance genes and virulence factors in the phage genome were predicted using the online platforms ResFinder (17) and VirulenceFinder (18), respectively. The whole-genome sequence of phage_SAP_1432 was analyzed using BLAST to identify closely related phage genomes.

### Growth curves of bacteria co-cultured with phage at different temperatures

For the growth curves of bacteria and phage co-cultures, we adapted the method described by Padfield et al. (13) with slight modifications. Based on the critical temperature of *S. aureus* 1432, eight temperatures (20°C, 30°C, 35°C, 37°C, 39°C, 42°C, 45°C, and 50°C) were selected. *S. aureus* 1432 cultures (10 µL, 10⁷ colony-forming units/mL) in the log phase were added to 32 wells of a 96-well plate. Phage suspensions (10 µL) were introduced at different MOIs (0.001, 0.1, and 100) into 24 of the 32 wells, followed by the addition of 180 µL of LB medium to each well. The remaining eight wells contained only *S. aureus* 1432 cultures without phage as bacterial control groups. As blank controls, 200 µL of LB medium was added to another eight wells without containing *S. aureus* 1432 and phage_SAP_1432. To minimize evaporation during incubation, a water-filled plate was placed above the 96-well plate. OD_600_ was measured every 3 h using a Microplate Absorbance Reader (Bio-Rad iMark). This experiment was repeated three times.

### Wrangling of data

The growth curve models were fitted by using the R package “nlsMicrobio” (19), and Akaike information criterion scores were calculated using the R package “broom” (20). The best-fitting TPC model with the smallest Akaike information criterion score was selected using the R package “rTPC” (21); based on this model, the bacterial growth rate, activation energy, and deactivation energy were calculated using the same R package. The Bayesian effect was simulated using the R package “brms” (22) to obtain the interval estimation.

### Detection of lysogens at different temperatures and phage concentrations

Bacterial hosts can survive beyond the lytic phase of phages through lysogeny after co-culture (23). The effects of temperature, co-culture duration, and phage concentration on lysogeny were evaluated by measuring the percentage of lysogens under different experimental conditions. Lysogens were confirmed through the amplification of specific phage_SAP_1432 genes, including integrase, holin, and amidase, from the bacterial genomic DNA. Primer sequences for these gene fragments were designed using Primer 5.0 software (Table S1) and synthesized by DynaScience Biological Company (Xi an, China). One-hundred microliters of mixed bacteria–phage suspension was collected at 0 and 33 h of incubation (MOI = 0.001, 0.1, and 100). After removing unabsorbed phages by centrifugation, we cultured the mixture on solid LB medium for 16–18 h. Subsequently, we selected 40 colonies from each solid medium to extract DNA for PCR amplification, determining the percentage of colonies that contained fragments of the phage_SAP_1432 holin, integrase, and amidase genes. This experiment was repeated three times.

### The impact of elevated temperatures and phage concentrations on the survival of *S. aureus* Q1432 in the co-culture system

Given that *S. aureus* Q1432 can be inactivated by culture at 51°C for 1 h, we assessed the impact of high temperatures and phage concentrations on the survival of the bacterium in a co-culture system with phage_SAP_1432. First, 1 mL of purified phage (10^9^ plaque-forming units/mL) was placed in a digital dry bath (Thermo Fisher Scientific Ltd.) at 80°C for 2 h. After heat treatment, the phages were cultured on double-layered plates to obtain surviving plaques, which were then amplified and purified for further analysis. Heat-treated phage_SAP_1432 culture was mixed with *S. aureus* Q1432 at various MOIs (0.001, 0.1, 100, and 10,000). These mixtures were incubated at a range of temperatures (55°C, 65°C, 70°C, and 80°C) for 2 h. The resulting mixtures were then plated on LB agar, and bacterial colony growth was observed on the subsequent day. To determine whether phage_SAP_1432 confers thermal tolerance on other *S. aureus* isolates, we selected three additional *S. aureus* hosts of phage_SAP_1432 for confirmation: *S. aureus* Q1622, *S. aureus* Q14261, and *S. aureus* Q14098. These strains were subjected to the same co-culture conditions with phage_SAP_1432 at different MOIs and temperatures. All experiments were performed in triplicate.

## RESULTS

### Characteristics of phage_SAP_1432

**Fig 1:**
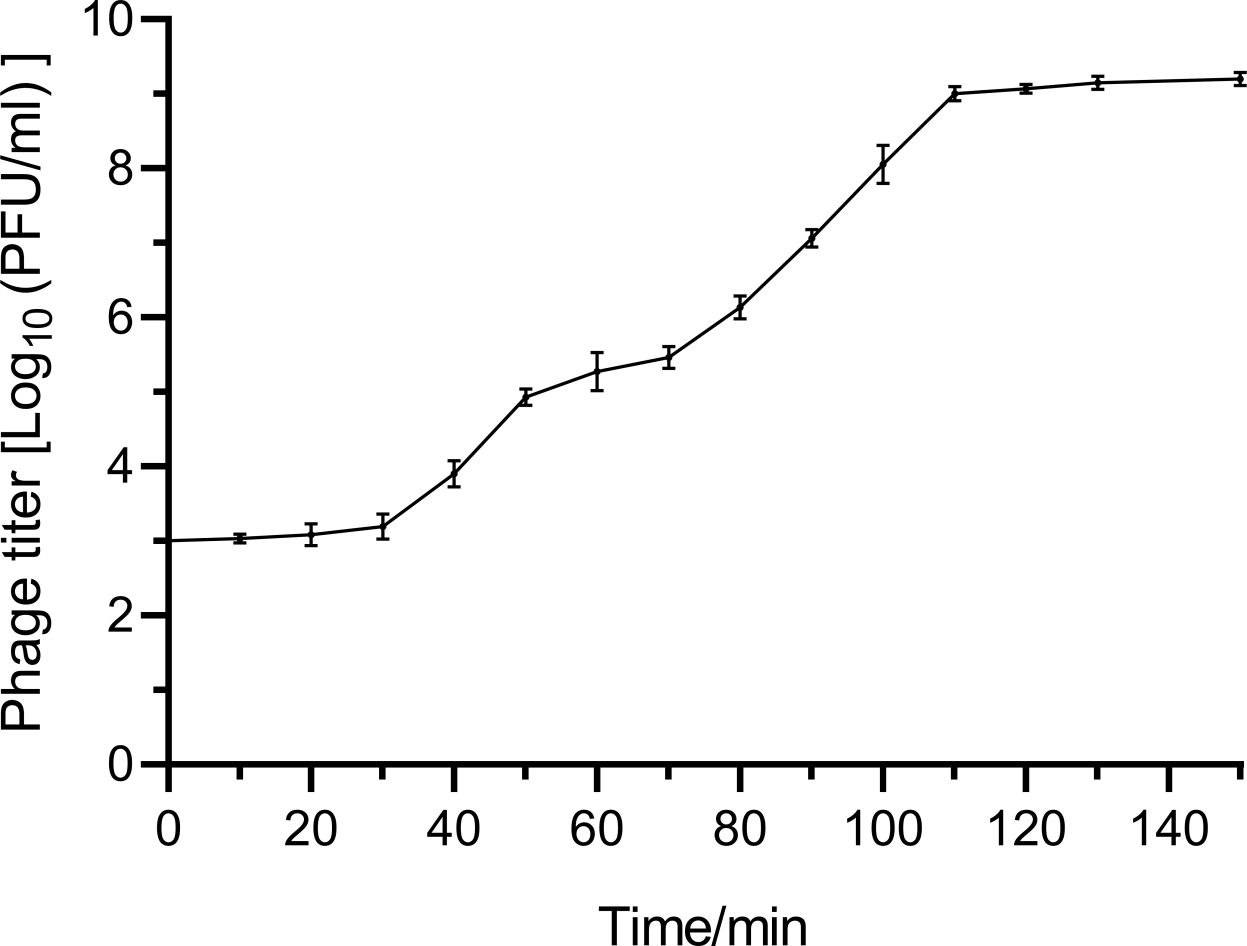
One-step curve of phage_SAP_1432.

**Fig 2:**
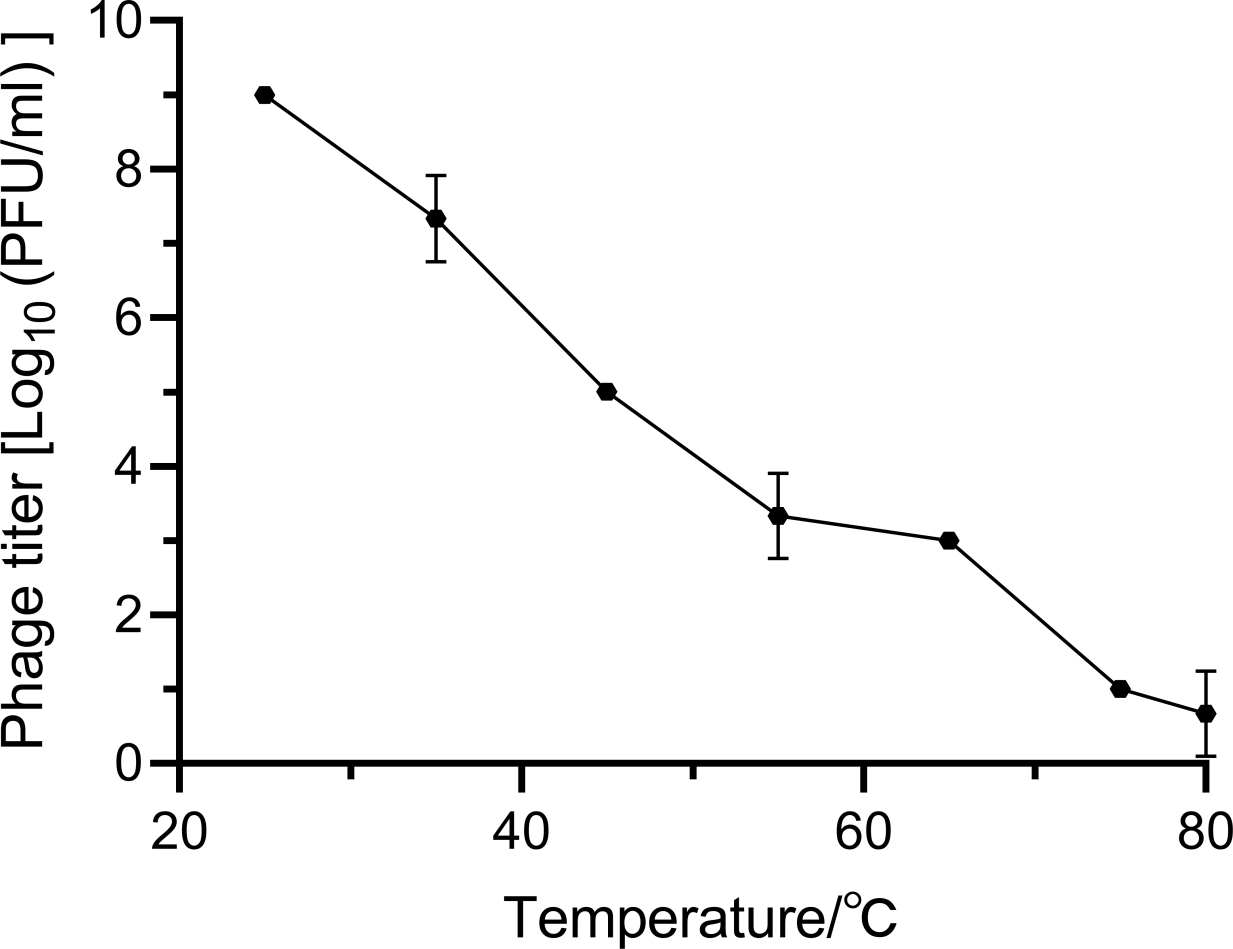
Thermal stability of phage_SAP_1432.

**Fig 3:**
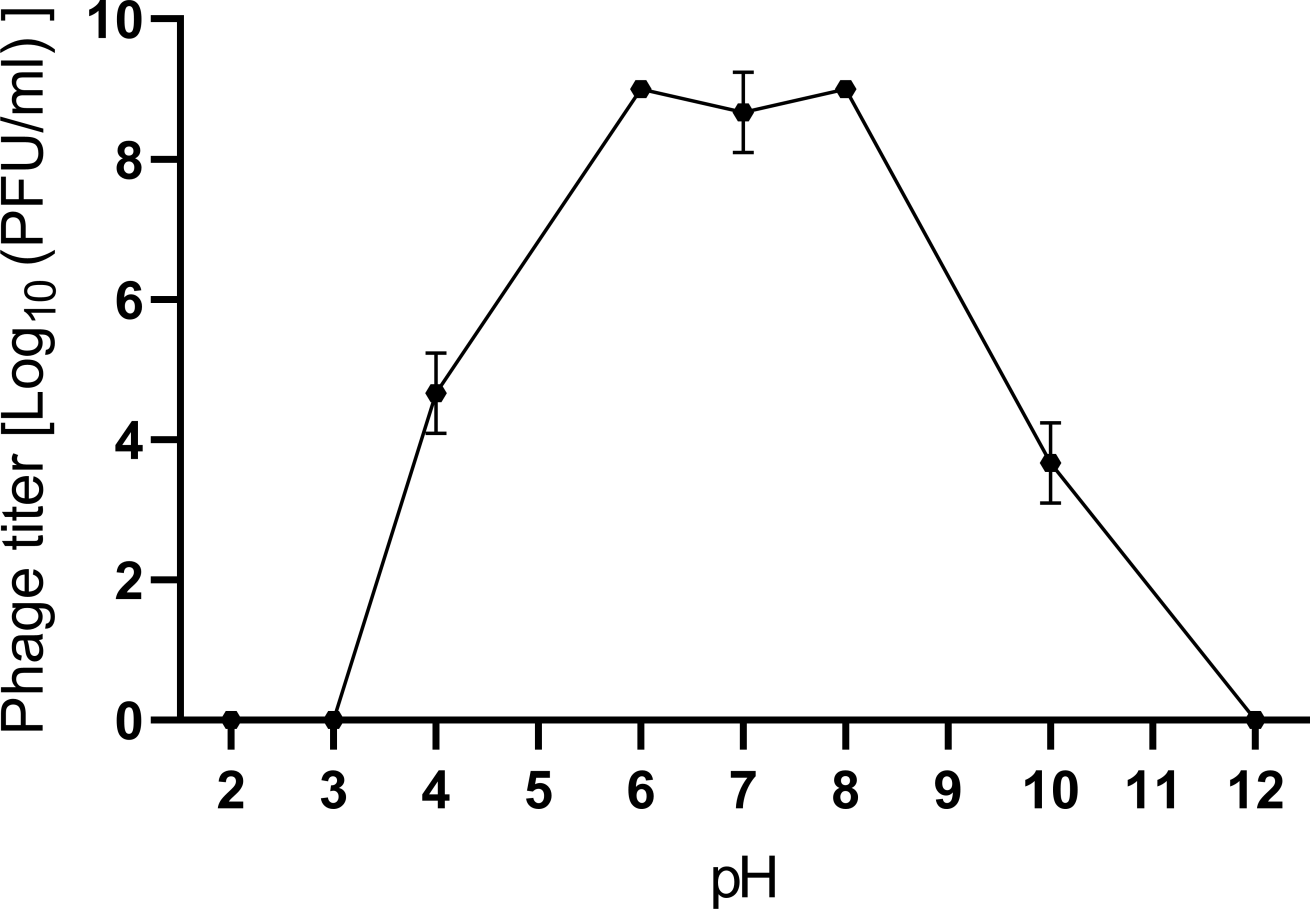
pH stability of phage_SAP_1432.

The optimal MOI for *S. aureus* Q1432 was 0.1. The one-step growth curve showed that the latent period and the burst period were 30 and 110 min, respectively (Fig. 1). As the measurement temperature increased, the activity of phage_SAP_1432 gradually decreased, but some phages still retained activity at 75–80°C, indicating that phage_SAP_1432 was heat-resistant (Fig. 2). Phage_SAP_1432 maintained activity at a neutral pH (pH 6.0-8.0) and showed resistance to alkaline (pH 8.0-12.0) and acidic conditions (pH 4.0–6.0) (Fig. 3).

### Genome sequencing and annotation of phage_SAP_1432

The complete genome of phage_SAP_1432 was sequenced, analyzed, and deposited in GenBank under accession number MZ570582. The genome of phage_SAP_1432 was 40,357 bp (G + C: 34.73 mol%). In total, 59 open reading frames were predicted, 25 of which encoded proteins with known functions. Annotation revealed the presence of an integrase gene, indicating that it is a temperate phage. Phage_SAP_1432 does not carry any virulence, drug resistance, or toxin genes in its chromosome. The BLAST results indicated that phage_SAP_1432 shares the closest genetic relationships with phage B_UFSM3 (MW627293.1), phage B_UFSM1 (MW650841.1), phage B_UFSM5 (MW192778.1), and phage B_UFSM4 (MW147366.1), with nucleotide identity exceeding 99%.

### Both the optimum temperature and maximum growth rate of *S. aureus* Q1432 increased in a bacteria–phage co-culture system

**Fig 4:**
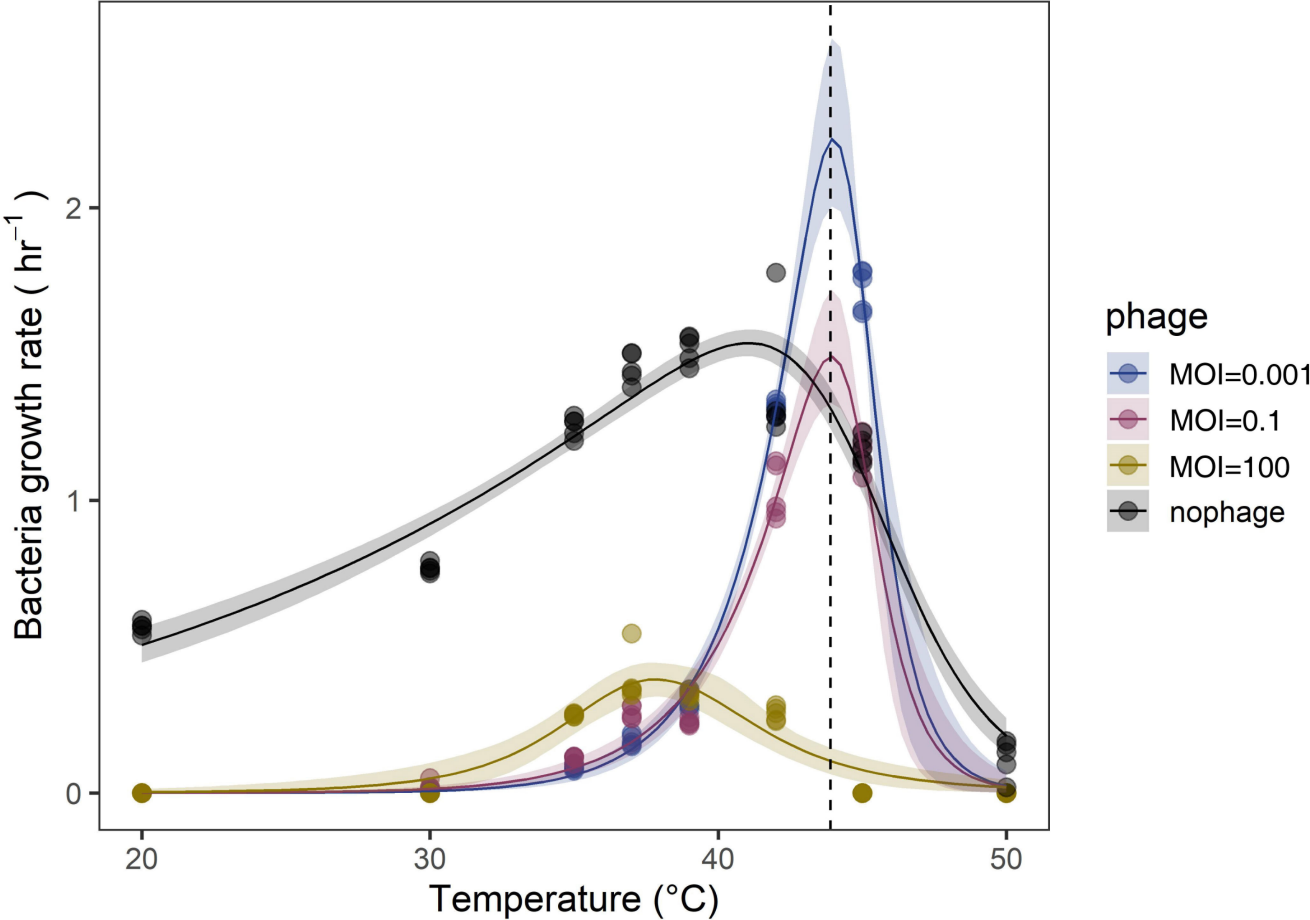
TPCs of *S aureus* Q1432 in the absence of phage and in the presence of phage_SAP_1432 with different MOIs.

We measured the growth rate of *S. aureus* Q1432 at eight temperatures (20°C–50°C) in the absence and presence of phage_SAP_1432. We modeled the TPC in each condition and used estimated and derived parameters of the models (Materials and Methods, Table S2) to compare the thermal responses of the bacterium. In the absence of phage_SAP_1432, the bacterial growth rate increased up to the thermal optimum temperature (T_opt_) of 41.0°C (95% confidence interval [CI]: 40.5°C–41.6°C) (Fig. 4). Phage_SAP_1432 (MOI = 0.001) increased the T_opt_ from 41.0 to 44.1°C (95% CI: 43.9°C–44.1°C) (Fig. 4) and resulted in a 41.2% increase in the maximal host growth rate from 1.53 (95% CI: 1.42–1.63) to 2.16 (95% CI: 2.02–2.37).

### The T_opt_ and maximum growth rate of the host bacterium is dependent on the phage concentration in the bacteria–phage co-culture system

**Fig 5:**
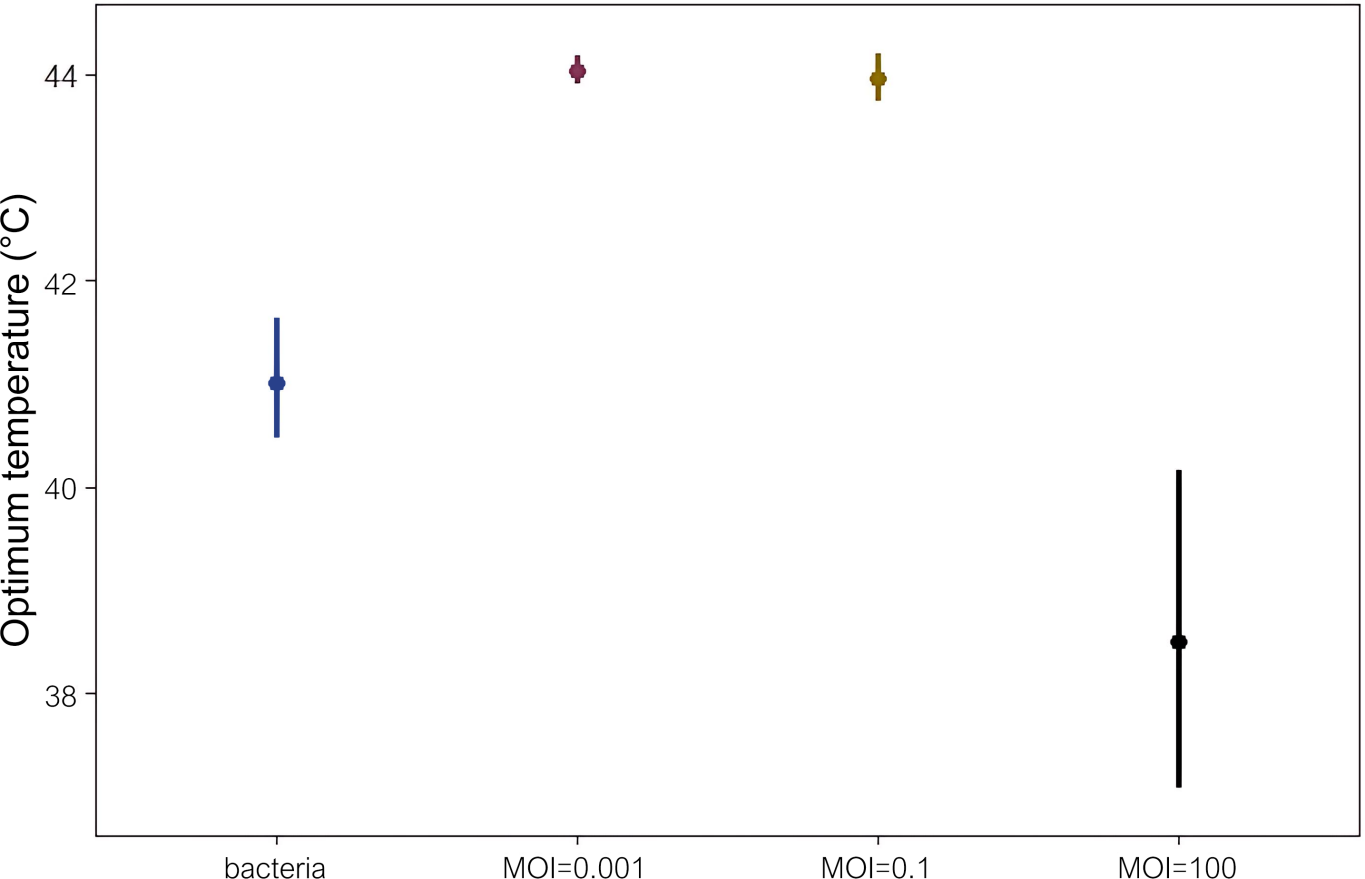
Prediction of optimum growth temperature of *S. aureus* Q1432 in the presence of phage_SAP_1432 at different MOIs and in the absence of phage (data labeled “bacteria”).

We increased the phage concentration and co-cultured phage_SAP_1432 and *S. aureus* Q1432 at different temperatures. When the MOI increased to 0.1, the T_opt_ of the host bacterium did not change compared with that at an MOI of 0.001, but the maximum growth rate decreased to 1.44 (95% CI: 1.32–1.71). At MOI = 100, T_opt_ of the host bacterium shifted to 38.4°C (95% CI: 37.1°C–40.9°C), and the maximum growth rate decreased to 0.38 (95% CI: 0.32–0.47) ((Fig. 5).

### The activation and deactivation energy of the bacterial host are dependent on the MOI

**Fig 6:**
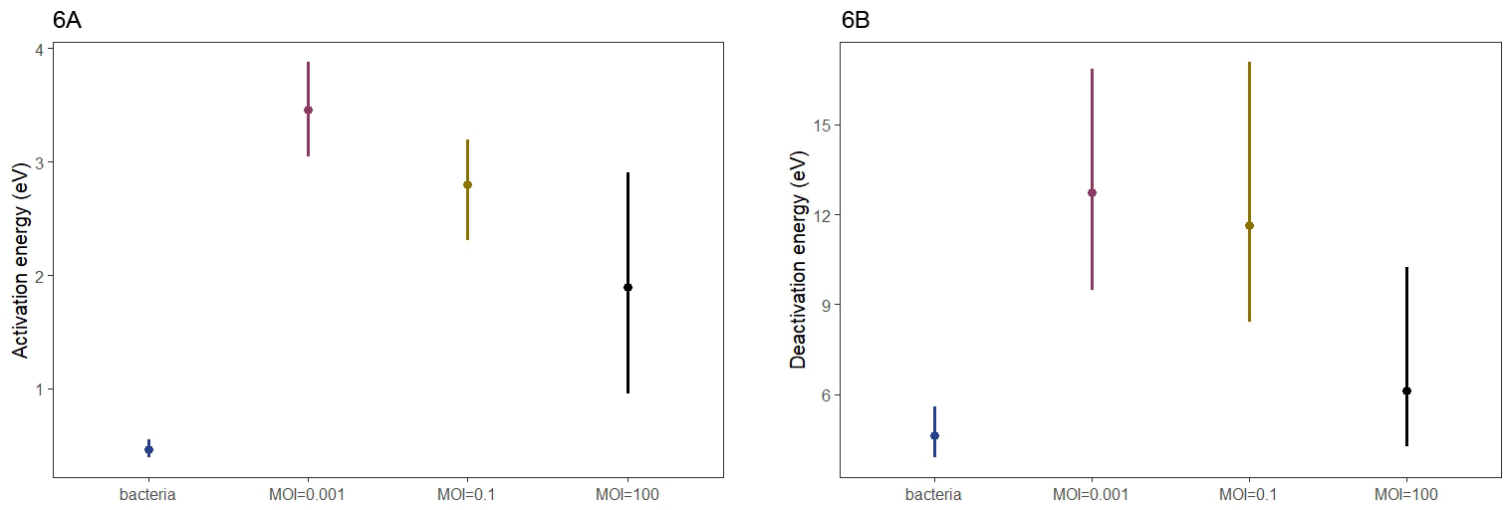
Prediction of activation and deactivation energy ranges of *S. aureus* Q1432 in the presence of phage_SAP_1432 at different MOIs and in the absence of phage (data labeled “bacteria”). (**A**) Prediction of activation energy. (**B**) Prediction of deactivation energy.

The changes in growth also resulted in differences in the activation and deactivation energy of the bacterial host (Fig. 6). In the absence of phage_SAP_1432, the activation energy was 0.27 (95% CI: 0.21–0.34) for *S. aureus* Q1432, increasing to 3.45 (95% CI: 3.07–3.81) at MOI = 0.001. As the MOI increased, the activation energy decreased (Fig. 6A). The deactivation energy of *S. aureus* Q1432 also decreased with increasing MOI (0.001–100) (Fig. 6B).

### The percentage of lysogens was affected by temperature and phage concentration

**Fig 7:**
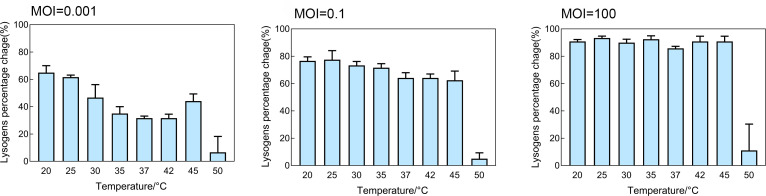
Percentage *S. aureus* Q1432 lysogens at different temperatures in the presence of different phage_SAP_1432 concentrations at 33 h.

Co-culture of the bacterium and phage_SAP_1432 for 33 h resulted in different percentages of lysogens across MOIs (Fig. 7). At MOI = 0.001, the percentage of lysogens was highest at 20°C and 25°C, gradually decreasing as temperature increased. At 50°C, lysogens were significantly reduced. At MOI = 0.1, the percentage of lysogens remained consistently high from 20°C to 45°C, showing only minor variation; a sharp decline was observed at 50°C, dropping below 10%. At MOI = 100, lysogens were near 100% from 20°C to 45°C, indicating that at high phage concentrations, nearly all bacterial cells were converted. However, at 50°C, lysogens dropped significantly to below 20%, suggesting that extreme temperatures limit phage integration despite MOI.

### Bacteria survived at high temperatures in the host–parasite co-culture system

**Fig 8:**
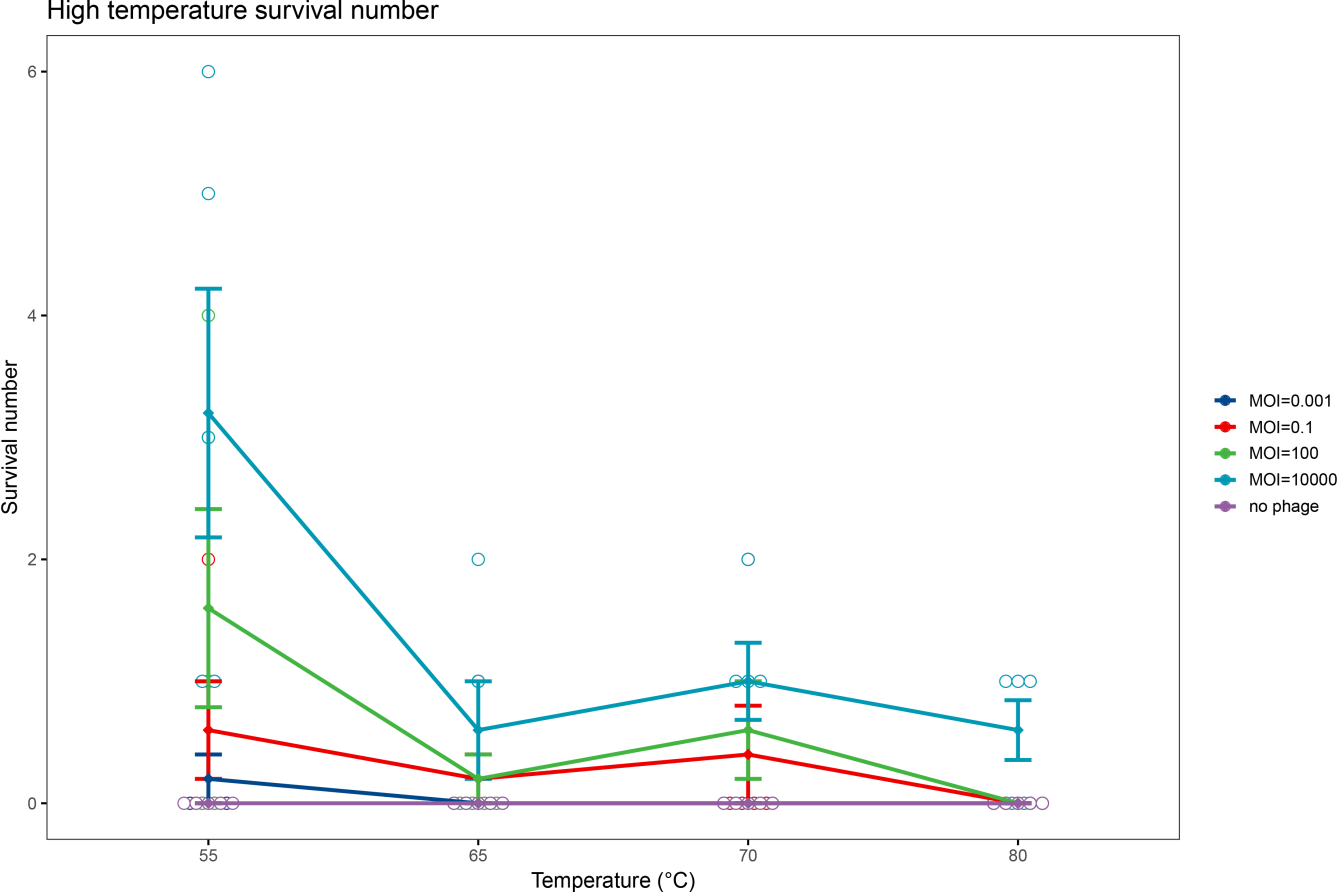
Survival numbers of *S. aureus* Q1432 at high temperatures with different MOIs of phage_SAP_1432.

We detected the effect of high temperatures (55°C, 65°C, 70°C, and 80°C) and phage concentrations on the survival of *S. aureus* Q1432 co-cultured with the heat-resistant temperate phage_SAP_1432 for 2 h. When the MOI was 0.001, a few *S. aureus* Q1432 clones were observed after co-culture at 55°C; however, no clones were detected when the co-culture temperature was ≥65°C. When the MOI was 0.1 or 100, a few *S. aureus* Q1432 clones could be observed at 55°C, 65°C, and 70°C, but no clones were detected after co-culture at 80°C. When the MOI was 10,000, *S. aureus* Q1432 clones could be observed after co-culture at 55°C, 65°C, 70°C, and 80°C (Fig. 8; Fig. S1). *S. aureus* Q1622, *S. aureus* Q14261, and *S. aureus* Q14098 all exhibited survival at >60°C after co-culture with phage_SAP_1432 at MOI = 100. All strains were found by PCR examination to carry the integrase, holin, and amidase genes of phage_SAP_1432 (Fig. S2 to S4).

## DISCUSSION

Climate change, especially global warming, has profound effects on bacterial communities across various ecosystems (24–26). Numerous studies have shown that temperature plays a crucial role in bacteria–phage co-evolution, altering the outcomes of phage–host interactions in complex ways (27, 28). TPCs are good indicators to predict the impacts of warming on host–parasite interactions (13, 29). In this study, the heat-resistant temperate phage changed the TPCs of its bacterial host. There were also some differences between the TPCs of the bacterial host in a lytic phage–bacterial host co-culture system and the heat-resistant temperate phage–bacterial host co-culture system. Co-culture of phage_SAP_1432 and its bacterial host *S. aureus* Q1432 (MOI = 0.001) led to an increased T_opt_ of the host, consistent with previous research (13). However, the TPCs revealed that the maximum growth rate and activation energy were higher than those of the bacterial host cultured alone, differing from previous research (13) in which co-culture of a lytic phage and its bacterial host resulted in a lower maximum growth rate and activation energy. However, when we increased the MOI of phage_SAP_1432 to 100, T_opt_ for the bacterium decreased to 37°C and the maximum growth rate also decreased. Kick et al.(30) reported that bacteriophage M13 infection led to a decrease in the maximum specific growth rate of 15% for *Escherichia coli* JM109. The observations at MOI = 0.001 might be attributed to the combined effects of sporadic phage infections, temperature-imposed growth constraints, and altered population dynamics. Our lysogeny assay confirmed that lysogeny was not the primary factor driving the increased bacterial growth. At low MOI, the phage-to-bacterium ratio was low, leading to infrequent infections, allowing a substantial portion of bacteria to continue dividing before encountering phages (31). Additionally, high temperatures reduce phage lysis efficiency (32), further stabilizing the bacterial population (33). While elevated temperatures also suppressed bacterial replication, the reduced phage predation allowed the co-cultured population to reach the stationary phase more quickly than monocultures, likely because of the combined effects of limited bacterial growth and weaker phage-mediated suppression. In contrast, at MOI = 100, nearly all bacterial cells encountered phages early in the culture, leading to significant growth inhibition. Despite high temperatures reducing phage lysis efficiency, the overwhelming number of infecting phages ensured that the inhibitory effect remained dominant, preventing bacterial population stabilization.

Lysogenesis was also more frequent at lower temperatures (<25°C), consistent with previous studies (28, 34, 35). This may be because lysogens are associated with low concentrations of susceptible hosts, which decrease the benefits of lysis for the phage (36). In addition to host density, temperature may directly influence the regulatory mechanisms that govern the lysogenic-lytic switch. Previous research has shown that temperature fluctuations can affect the expression of key phage genes, including those involved in the switch between lysogeny and lysis (37, 38).

The influence of temperature on lysogeny is particularly relevant in the context of *S. aureus*, a globally prevalent and opportunistic pathogen. Because of its increasing resistance to antibiotics, research on *S. aureus* and its interactions with phages has gained considerable attention (39–45). In recent years, studies have been conducted that aim to use lytic phages to eliminate *S. aureus*. However, before temperate phages can be applied, it is necessary to understand the proportion of lysogens in the host population in different circumstances. In this study, co-culture of the phage and its host at high temperatures (55°C–80°C) resulted in some, albeit few, lysogens. This is consistent with previous studies showing that phages, such as lactococcal phages and those infecting *Salmonella enteritidis*, could survive at high temperatures in broth and milk (46–50). The survival of lysogens in pasteurized environments may lead to bacterial persistence and potential food contamination, increasing the risk of outbreaks caused by heat-resistant *S. aureus* strains (51).

Taken together, phage_SAP_1432 not only affected the thermal performance and growth rate of its host bacterium but also changed its characteristics through lysogeny. Our study illustrates the great adaptability of the phage–bacterial host system when confronted with changing temperatures. From a clinical and environmental perspective, temperate phages enhancing bacterial thermal tolerance may impact pathogen survival in high-temperature conditions, including fever, disinfection, and pasteurization. Additionally, phages could stabilize microbial communities under global warming, influencing pathogen persistence and thermal sterilization efficacy. However, our study had some limitations, as we did not sequence the host bacterial genome or extend the experimental duration, limiting our ability to assess long-term phage–host interactions under varying temperature conditions. Future research should address these aspects to further elucidate the evolutionary dynamics of heat-resistant temperate phages and their bacterial hosts.

## Data Availability

All data and findings from this study are included in the article. For additional information, please contact the corresponding author.

## SUPPLEMENTAL MATERIAL

Tables S1 to S2, and Figures S1 and S2 spectrum.02271-24-s0001.docx Primers of related genes, AIC scores of Sharpeschoolfieldhigh model, and PCR results of phage_SAP_1432 genome identification.

ASM does not own the copyrights to Supplemental Material that may be linked to, or accessed through, an article. The authors have granted ASM a non-exclusive, world-wide license to publish the Supplemental Material files. Please contact the corresponding author directly for reuse.
