# Supplementary material for: Frenemy: adaptive temperate phage_SAP_1432 supports Staphylococcus aureus survival in changing temperatures
Source: Microbiol Spectr. 2025 Jun 23;13(8):e02271-24. doi: 10.1128/spectrum.02271-24 (PMC12323586; doi:10.1128/spectrum.02271-24)
Supplement: Tables S1 to S2, and Figures S1 and S2 — Primers of related genes, AIC scores of Sharpeschoolfieldhigh model, and PCR results of phage_SAP_1432 genome identification. [file spectrum.02271-24-s0001.docx]

Supplementary Material

Frenemy: Adaptive Temperate Phage_SAP_1432 Supports *Staphylococcus aureus* Survival in Changing Temperatures

**Ting-ting Liu, Peng-cheng Gao, Jie-wen Cui, Wu-bin Wang, Fu-ying Zheng, Xue-rui Li^*^, Yue-feng Chu^*^**

*** Correspondence:**

Xue-rui Li
[lixuerui@caas.cn](mailto:lixuerui@caas.cn)

Yue-feng Chu
[chuyuefeng@caas.cn](mailto:chuyuefeng@caas.cn)

# Supplementary Tables

# Table S1 Primers of related genes

| - Primer | Primer Sequence |
| --- | --- |
| Amidase-F | ATGTTAATGACAAAAAATCAAGCAG |
| Amidase-R | CTACAAAAGGCTGAATTTCCCAAAG |
| holin-F | ATGGAAGCAAAAGTAATAACAAGAT |
| holin-R | CTACCCTAAATCATTTGTATCGTTC |
| Intergrase-F | ATGAAAGTAGCAATTTATACCAGAG |
| Intergrase-R | ATAAAACTCTATACCAGTAATCTTC |

# Table S2 AIC scores of Sharpeschoolfieldhigh model

| Sharpeschoolfieldhigh | AIC scores |
| --- | --- |
| Bacteria alone | -44.4 |
| MOI=0.001 | -148.2 |
| MOI=0.1 | -145.7 |
| MOI=100 | -111.6 |

# Supplementary Figures

# Figure S1 Results of phage_SAP_1432 genome identification


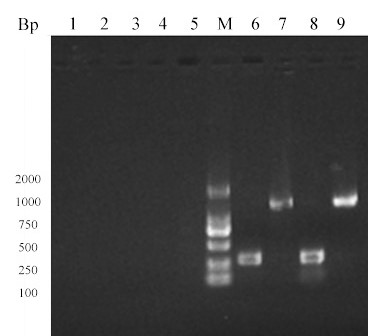


M is DNA marker, 1-5 are amplification results of related genes without co-culture; 6,8 are amplification results of bacterial holin genes surviving at high temperature, 7 are Intergrase genes amplification results of bacterial surviving at high temperature, 9 are Amidase genes amplification results of bacterial surviving at high temperature.

#
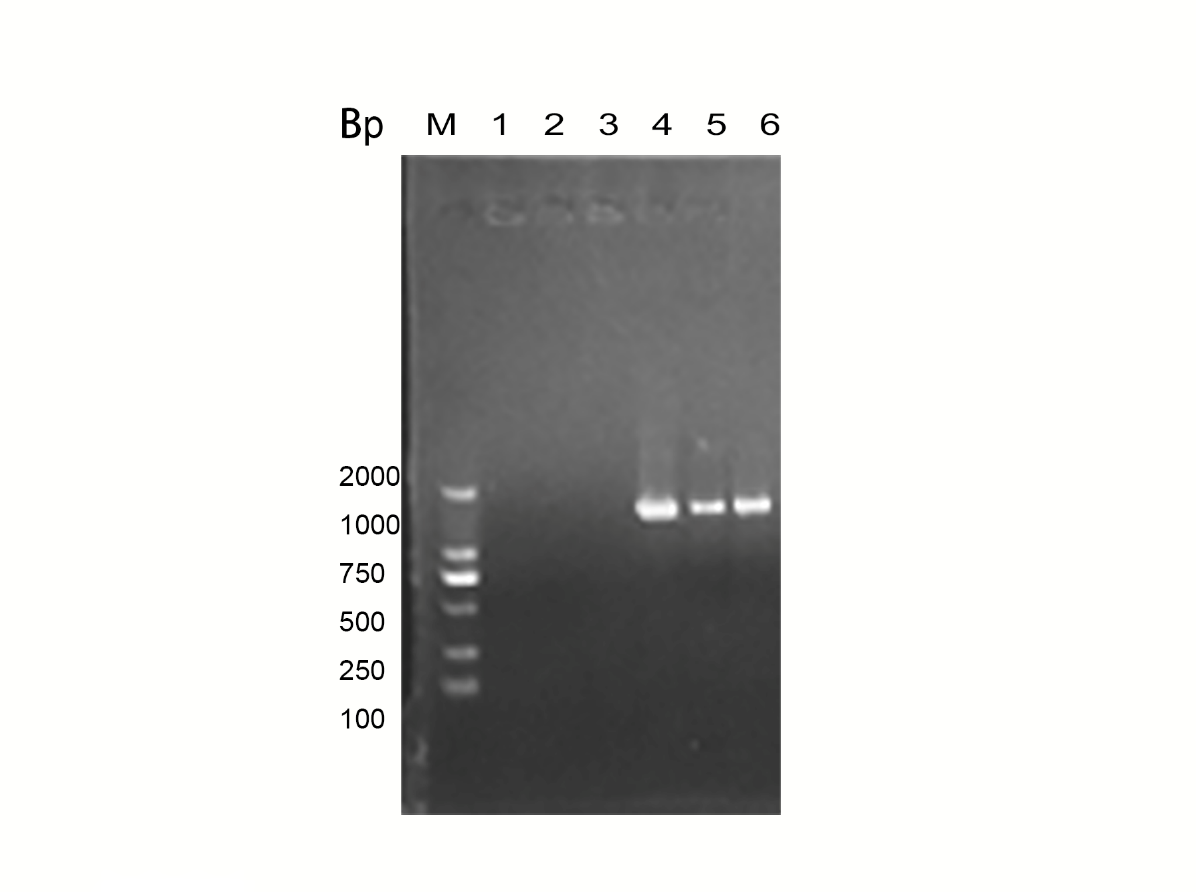
 Figure S2 Results of Integrase gene identification in *S. aureus* Q1622, *S. aureus* Q14261, and *S. aureus* Q14098

M is DNA marker, 1-3 are amplification results of Integrase gene without co-culture (*S. aureus* Q1622, *S. aureus* Q14261, and *S. aureus* Q14098); 4-6 are amplification results of Integrase gene with co-culture.

# Figure S3 Results of holin genome identification in *S. aureus* Q1622, *S. aureus* Q14261, and *S. aureus* Q14098


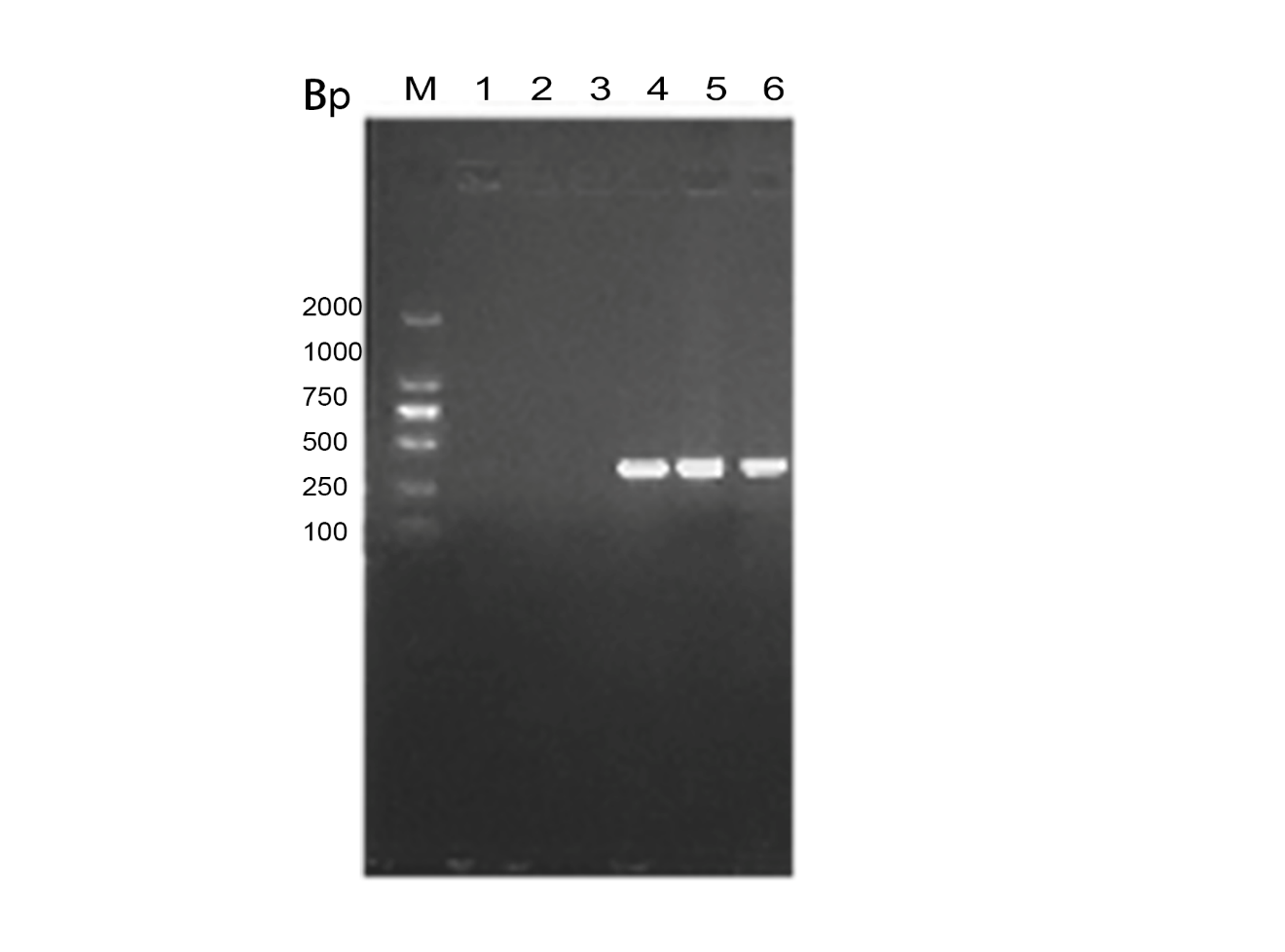

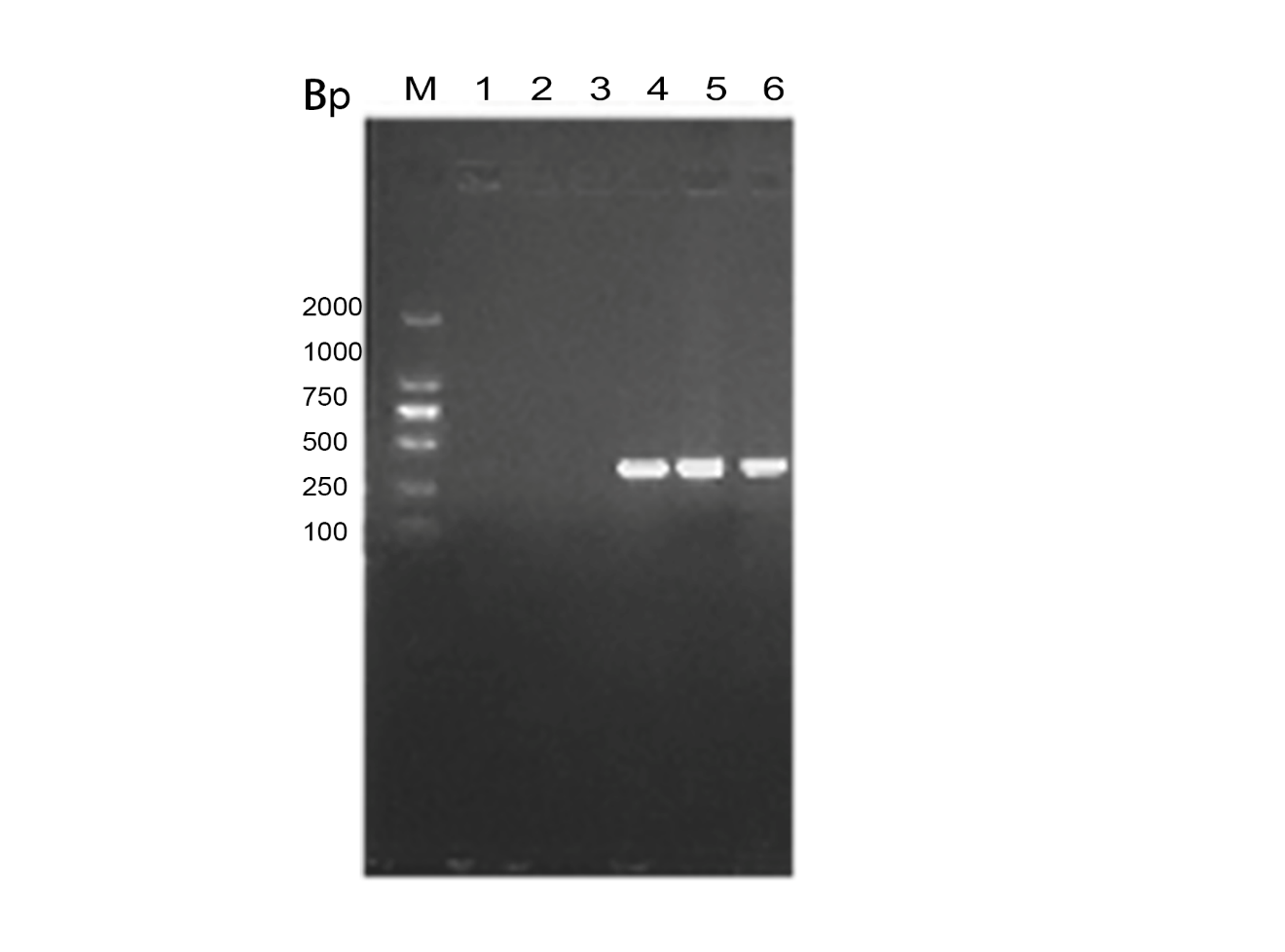


M is DNA marker, 1-3 are amplification results of holin gene without co-culture (*S. aureus* Q1622, *S. aureus* Q14261, and *S. aureus* Q14098); 4-6 are amplification results of holin gene with co-culture.

# Figure S4 Results of amidase gene identification in *S. aureus* Q1622, *S. aureus* Q14261, and *S. aureus* Q14098


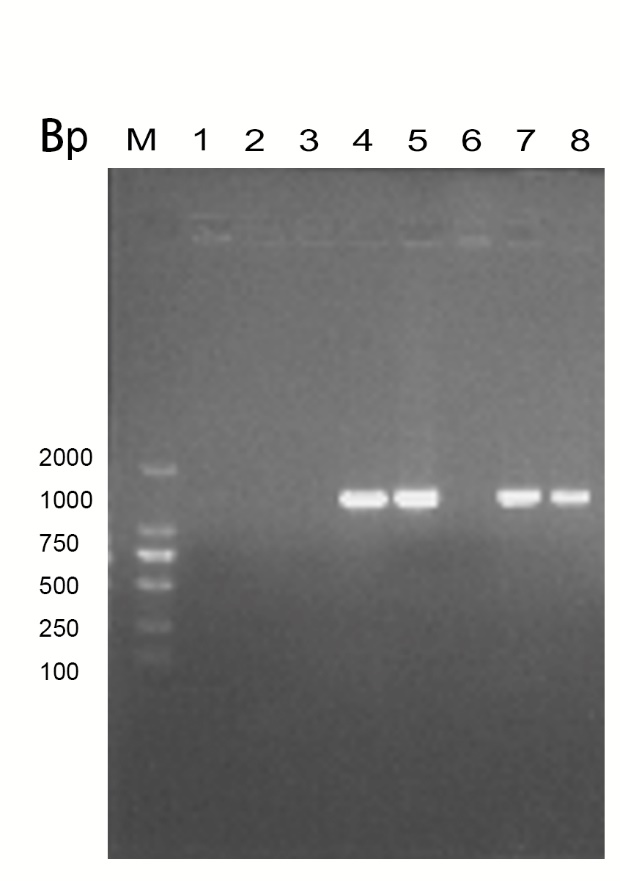


M is DNA marker, 1-3, 6 are amplification results of amidase gene without co-culture (*S. aureus* Q1622, *S. aureus* Q14261, and *S. aureus* Q14098); 4, 5, 7,8 are amplification results of amidase gene with co-culture.
